# Red meat vs. white meat: A comparative analysis of histological characteristics, nutritional profiles, and health implications

**DOI:** 10.1016/j.fochx.2025.103211

**Published:** 2025-10-26

**Authors:** Huiling Zhang, Mailin Gan, Qihang Wu, Haifeng Dan, Lei Chen, Lili Niu, Ye Zhao, Yan Wang, Li Zhu, Linyuan Shen

**Affiliations:** a Farm Animal Genetic Resources Exploration and Innovation Key Laboratory of Sichuan Province, Sichuan Agricultural University, Chengdu 611130, China; b State Key Laboratory of Swine and Poultry Breeding Industry, Sichuan Agricultural University, Chengdu 611130, China; c Key Laboratory of Livestock and Poultry Multi-omics, Ministry of Agriculture and Rural Affairs, College of Animal and Technology, Sichuan Agricultural University, Chengdu 611130, China

**Keywords:** Red meat, White meat, Heme iron, Fatty acids, Trimethylamine N-oxide, Chronic diseases

## Abstract

This review critically assesses the nutritional and health impacts of red versus white meat. Current evidence strongly associates red meat consumption (beef, pork, lamb) with increased risks of colorectal cancer, cardiovascular disease, and type 2 diabetes, primarily attributed to its high heme iron and saturated fat content. In contrast, white meat (poultry, seafood) demonstrates a more favorable risk profile and may be protective. By synthesizing the current biochemical evidence, this review highlights the contrasting health effects of both meat types. While red meat consumption is associated with increased health risks, moderate intake of white meat appears to offer a healthier alternative. Based on these findings, the review provides evidence-based dietary recommendations to inform consumer choices and help mitigate associated health risks.

## Highlights

• Compared the histological and nutritional profiles of red and white meat, analyzing the factors influencing these components.
• Assessed the benefits and risks of red and white meat consumption.
• Investigated how components like heme iron and saturated fatty acids induce disease.
• Provided evidence-based dietary recommendations for balancing the intake of red and white meat.

## 1 Introduction

Meat has been a staple in the human diet for millions of years, playing a crucial role in human evolution, with evidence suggesting our ancestors began incorporating it into their diets as early as 26 million years ago (Milton, 2003). Meat is primarily composed of water (60–80 %), protein (16–25 %), and fat (1–30 %), along with trace amounts of non-protein nitrogenous compounds, carbohydrates, vitamins, and minerals. In recent decades, both global meat consumption and production have risen significantly. The Food and Agriculture Organization (FAO) projects a 76 % increase in total meat consumption by mid-century, with poultry consumption expected to double, beef rising by 69 %, and pork by 42 %. As meat preferences shift, poultry is increasingly replacing beef and processed meats as the primary protein source.

Considerable research suggests that the consumption of various types of meat and their derived products has a profound effect on human health. In academic discussions, the terms “red meat” and “white meat” are frequently used to categorize different meats. The International Agency for Research on Cancer (IARC) defines red meat as the fresh, unprocessed muscle tissue of mammals. Common examples include beef, veal, pork, lamb, goat meat, and horse meat (Bouvard et al., 2015). The European Prospective Investigation into Cancer and Nutrition (EPIC) classifies poultry as white meat, with some epidemiological studies also including fish in this category (Rohrmann et al., 2013). Though certain meats, like salmon and poultry legs or wings, may appear red, they are still classified as white meat. The red color of salmon primarily stems from its high carotene content, which is unrelated to myoglobin, so this does not alter salmon's classification as white meat. Similarly, while some poultry muscles, like those in the legs and wings, have a reddish appearance due to higher myoglobin concentrations, they are still uniformly categorized as white meat. Processed meat refers to meat that has been preserved through methods such as pickling, air-drying, fermentation, smoking, or the addition of chemical preservatives. This category includes products such as sausages, bacon, ham, and cooked meats, and is distinct from both red meat and white meat.

The World Cancer Research Fund (WCRF) recommends limiting red meat intake to no more than 80 g per day, with processed meat intake kept to a minimum. Subsequent guidelines further reduced this limit to 71 g per day or 500 g per week, emphasizing that processed meat should be avoided entirely (Mcafee et al., 2010). Numerous prospective studies and meta-analyses from high-income countries provide strong evidence that high consumption of red and processed meats significantly increases the risk of cardiovascular diseases and certain cancers (Farvid et al., 2021; Forouzanfar et al., 2015; Kaluza et al., 2012; Mcafee et al., 2010; Misra et al., 2018; Omaye & Omaye, 2019). A case-control study by Deoula et al. found a positive association between red and processed meat intake and colon cancer risk, with no significant link observed for white meat (Deoula et al., 2020). A cohort study in the U.S. by Ma et al. showed that higher red meat consumption was associated with an 84 % increased risk of hepatocellular carcinoma, while white meat was linked to a 39 % reduced risk (Ma et al., 2019). Additionally, red meat consumption is associated with a higher risk of breast cancer, whereas poultry may lower this risk (Lo et al., 2020). Overall, substantial evidence suggests that high consumption of red and processed meats increases mortality, cancer incidence, and cardiovascular diseases, while higher white meat intake is associated with lower health risks, including reduced mortality and cancer deaths.

This review offers a comprehensive and systematic evaluation of current evidence regarding the differences between red and white meat in terms of appearance, muscle fiber composition, and nutritional properties. Distinct from previous studies that often focused on isolated health outcomes or specific meat categories, our work adopts a mechanistic perspective that integrates intrinsic meat characteristics with compounds generated during cooking and processing. By mapping these factors onto pathways of disease pathogenesis, we uncover underexplored biological mechanisms underlying meat-related health risks. This integrative approach bridges the gap between nutritional science and molecular disease research, addressing a critical void in the existing literature. Our aims are twofold: (1) to elucidate the mechanistic basis for the differential health effects of red versus white meat, and (2) to translate these insights into evidence-based recommendations for health promotion and disease prevention.

## 2 Red meat vs. white meat

### 2.1 color

The color of meat is primarily determined by the myoglobin content in the muscle. Myoglobin is an iron-rich protein responsible for storing and transporting oxygen. Red meat contains higher concentrations of myoglobin, giving it a distinct red color. In contrast, poultry and most fish have relatively low myoglobin content, which is why their meat appears white or lighter in color.

### 2.2 Muscle fiber types

Muscle fibers can be classified into two primary types based on their contraction speed and metabolic characteristics: fast-twitch and slow-twitch fibers. Slow-twitch fibers rely mainly on oxidative metabolism for energy, which is why they are termed oxidative slow-twitch fibers (Type I). Fast-twitch fibers are further divided into two categories: the oxidative-glycolytic fast-twitch fibers (Type IIA), which possess both strong aerobic and anaerobic metabolic capabilities, and the glycolytic fast-twitch fibers (Type IIB), which mainly rely on glycolysis for energy, making them ideal for high-intensity, short-duration activities. Recent studies have also discovered a fourth category—glycolytic ultrafast-twitch fibers (Type IIX), which contract even more rapidly and have lower endurance (Lee et al., 2010). These muscle fiber types differ significantly in their myosin heavy chain (MyHC) isoform composition, oxidative metabolic capacity, and ATP production efficiency (Bassel-Duby & Olson, 2006). Slow-twitch fibers are rich in myoglobin and mitochondria, supporting sustained aerobic metabolism, and their high aerobic enzyme activity contributes to greater endurance in prolonged activities (Reyes et al., 2015). In contrast, fast-twitch fibers contain fewer mitochondria and primarily rely on anaerobic glycolytic metabolism, which makes them less endurance-oriented and more suited to short, high-intensity, explosive movements (Booth et al., 2015).

**Fig. 1:**
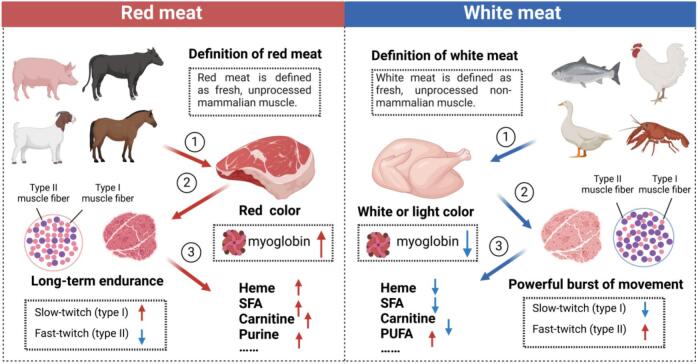
The differences between red meat and white meat. (For interpretation of the references to color in this figure legend, the reader is referred to the web version of this article.)

The various muscle fiber types described earlier are present in most muscles, and their relative proportions in different muscles influence the muscle's metabolic properties. In red meat, slow-twitch fibers make up a significantly larger proportion compared to white meat, enabling prolonged energy expenditure over extended periods. In contrast, white meat contains a higher percentage of fast-twitch fibers, which are better suited for quick, short bursts of intense activity (Fig. 1).

### 2.3 Nutritional components

#### 2.3.1 Amino acids

Meat contains high-quality proteins that provide a range of essential amino acids, including the eight required by adults and the nine needed by children. In terms of Protein Digestibility-Corrected Amino Acid Score (PDCAAS), meat typically scores around 0.9, indicating that its amino acids are of high quality and highly digestible. In comparison, most plant-based proteins score around 0.6, suggesting that their amino acid composition and bioavailability are lower (Williams, 2007). For every 100 g of raw red meat, there are typically 20–25 g of protein, with chicken and fish also falling within this range. Chicken can contain up to 20 % protein, while fish generally exceeds 15 %.

While both red and white meats are complete protein sources, subtle differences exist in their amino acid profiles. Red meat (e.g., beef) is particularly renowned for its high content of leucine, a branched-chain amino acid (BCAA) that plays a critical role in stimulating muscle protein synthesis(Layman et al., 2008). Conversely, white meats, especially chicken and fish, often contain slightly higher proportions of certain other essential amino acids like histidine and lysine. Fish, for instance, is also an excellent source of the amino acids arginine and taurine, which have various metabolic and cardiovascular benefits(Andersen et al., 2016).

#### 2.3.2 Fatty acids

Meat is not only a rich source of protein but also provides a significant amount of fatty acids. Red meat generally contains higher total fat content than white meat, with pork being particularly high in fat, followed by lamb and beef. The fat in meat consists of saturated fatty acids (SFAs), monounsaturated fatty acids (MUFAs), and polyunsaturated fatty acids (PUFAs). In ruminant animals like lamb and beef, the composition of fatty acids tends to have a higher proportion of saturated fats, which is closely linked to the hydrogenation process carried out by microbes in their gastrointestinal tract. Additionally, grass-fed animals have a higher proportion of omega-3 PUFA in their meat compared to grain-fed animals (Nogoy et al., 2022). In contrast, white meat typically contains lower levels of fat but has a higher proportion of unsaturated fatty acids, especially in fish. The unsaturated fats in fish can make up 10–20 % of the total fat content, with deep-sea fish being particularly rich in eicosapentaenoic acid (EPA) and docosahexaenoic acid (DHA).

#### 2.3.3 Vitamins and minerals

Red and white meat are also rich in various essential minerals, and compared to plant-based foods, the minerals in meat have higher bioavailability. Among these minerals, red meat stands out particularly for its iron and zinc content, making it an important source of these two minerals. For example, 100 g of beef contains approximately 3.3 mg of iron and 4.73 mg of zinc, while the same amount of chicken contains only 1.4 mg of iron and 1.09 mg of zinc.

In addition to minerals, meat is also rich in various water-soluble vitamins, especially B vitamins. Generally, red meat tends to contain higher levels of several B vitamins compared to white meat. Lean beef, for instance, contains about 2.5 micrograms of vitamin B_12_ per 100 g, which covers two-thirds of the daily requirement for adults. In comparison, chicken meat contains approximately 0.6 micrograms of vitamin B_12_ per 100 g. In addition to vitamin B_12_, meat is also rich in riboflavin (vitamin B_2_), niacin (vitamin B_3_), vitamin B_6_, and pantothenic acid, all of which play important roles in energy metabolism, immune function, and skin health (Cosgrove et al., 2005) (Table 1).

**Table 1:** Comparison of differences in appearance, nutritional composition, and cooking/processing by-products between red meat and white meat.

Category	Red Meat	White Meat	Ref.
Color	Redder due to higher myoglobin content, resulting in a darker color.	Lighter in color due to lower myoglobin content.	(Suman & Joseph, 2013)
Muscle fiber types	Predominantly composed of slow-twitch fibers, supporting endurance and sustained activity.	Contains a higher proportion of fast-twitch fibers, designed for rapid, short bursts of movement.	(Lee et al., 2010; Reyes et al., 2015)
Amino acids	Rich in essential amino acids with a Protein Digestibility-Corrected Amino Acid Score (PDCAAS) of around 0.92, making it an excellent source of complete protein.	Also contains high-quality proteins but typically with a higher PDCAAS (around 0.96), similar to red meat, supporting muscle repair and growth.	(Williams, 2007; Wyness, 2016)
Fatty acids	Generally higher in total fat content, particularly saturated fats, contributing to increased caloric intake.	Lower in total fat, with a higher proportion of unsaturated fats, which are considered healthier for cardiovascular health.	(Bhatt et al., 2019; Nicholls et al., 2020)
Vitamins and minerals	High in essential nutrients such as iron, zinc, and B vitamins (notably B_12_ and niacin).	Contains fewer amounts of iron and zinc but is rich in B vitamins, especially niacin, and tends to be higher in phosphorus.	(Cosgrove et al., 2005)
Bioactive compounds	Contains more bioactive compounds such as conjugated linoleic acid (CLA) and creatine, which have been linked to various health benefits, including muscle growth and fat reduction.	Fewer bioactive compounds in comparison to red meat but still a valuable source of nutrients such as choline and omega-3 fatty acids (especially in poultry).	(Liang et al., 2024; Wang et al., 2019)
Cooking/processing byproducts	May produce harmful compounds such as heterocyclic amines (HCAs) and polycyclic aromatic hydrocarbons (PAHs) when cooked at high temperatures, which are linked to cancer risk.	Fewer harmful byproducts compared to red meat, although processing (such as in the case of poultry) can still lead to some unhealthy compounds, particularly in processed meats.	(John et al., 2011)

### 2.4 Other bioactive compounds

In addition to traditional nutrients, meat also contains many important bioactive compounds that have unique effects on human health. Common bioactive substances found in meat include taurine, carnitine, carnosine, creatine, choline, purines, N-glycolylneuraminic acid (Neu5Gc), and glutathione. Notably, red meat generally contains higher levels of purines than white meat. For instance, beef typically contains around 110–130 mg of purines per 100 g, whereas chicken contains approximately 50–75 mg per 100 g. Carnitine, a key bioactive compound in red meat, plays a critical role in fatty acid metabolism. Beef contains approximately 80–100 mg of carnitine per 100 g, while chicken contains only 3–5 mg per 100 g. Carnitine is metabolized through the gut microbiota and the portal vein-liver circulation, eventually forming trimethylamine-N-oxide (TMAO). Studies have shown that red meat consumption is more likely to increase plasma TMAO concentrations compared to white meat and plant-based diets, with TMAO concentrations typically being three times higher in plasma after red meat consumption than after white meat intake (Wang et al., 2019). Additionally, red meat is rich in Neu5Gc, a derivative of sialic acid found in certain mammals. Although humans cannot synthesize Neu5Gc due to a genetic mutation, it can be acquired through the diet, particularly from red meat and dairy products. The concentrations of Neu5Gc in beef, lamb, and pork range from 25 to 231 μg/g, 8.83–97.27 μg/g, and 7–67.49 μg/g, respectively (Liang et al., 2024).

### 2.5 Cooking/processing byproducts

Different cooking and processing methods significantly impact the formation of carcinogenic byproducts. High-temperature grilling, open-flame cooking, and smoking all promote the production of harmful compounds such as heterocyclic amines (HCAs) and polycyclic aromatic hydrocarbons (PAHs) in meat (John et al., 2011). In contrast, methods such as stewing and steaming produce minimal or no carcinogens. Red meat and white meat exhibit significant differences in the mechanisms and types of carcinogen formation due to variations in fat content, myoglobin structure, and nutritional composition. At high temperatures (above 150 °C), reactions occur between creatine, amino acids, and sugars in the meat, leading to the formation of HCAs. Red meat, with its higher creatine content, generates more HCAs. Although white meat contains less creatine, it can still produce HCAs when subjected to high-temperature frying for extended periods. Additionally, when fat drips onto a heat source (such as an open flame or charcoal), smoke is generated, and the PAHs in the smoke adhere to the surface of the meat. Red meat, due to its higher saturated fat content, experiences more frequent dripping, resulting in significantly higher concentrations of PAHs compared to white meat.

Processed meat often contain elevated concentrations of nitrates and nitrites, which are commonly used as preservatives during processing. However, when nitrites and nitrates react with amines in the body, they can form carcinogenic N-nitroso compounds (NOCs). Furthermore, carnitine, a precursor to trimethylamine, may also facilitate the formation of NOCs. Even more concerning is that the heme iron in red meat can act as a catalyst under certain conditions, promoting the reaction between nitrites and amines. The accumulation of NOCs is closely linked to the development of various cancers, including gastric and colorectal cancers (Portha et al., 1980). Once these harmful chemicals enter the body, they can be metabolized into more reactive carcinogenic forms, ultimately leading to genetic mutations and the potential onset of cancer.

### 2.6 Health impact

Both red meat and white meat are nutrient-rich foods, yet they differ in their composition of proteins, fatty acids, minerals, and vitamins, each offering unique advantages. For instance, red meat is a significant source of heme iron, which helps prevent iron-deficiency anaemia, making it especially beneficial for pregnant women, adolescents, and the elderly. Studies suggest that moderate consumption of red meat can lower the risk of depression, possibly due to vitamin B's role in regulating neurotransmitters (Nucci et al., 2020). On the other hand, white meat features an amino acid profile that closely aligns with human nutritional needs and boasts a higher proportion of unsaturated fatty acids, which are protective against cardiovascular disease. Individual and population differences, such as age, gender, and dietary patterns, play a crucial role in moderating the health effects of meat consumption. Firstly, age-related factors lead to variations in the digestion and absorption of meat, as well as differing nutritional requirements across different age groups. For example, older adults may need to reduce their intake of red meat due to a slower metabolism, which can help lower the risk of cardiovascular diseases. In contrast, younger individuals often require higher protein intake to support growth and physical activity. Secondly, gender differences significantly impact the health effects linked to meat consumption. Studies show that men typically consume more red meat, which can increase their risk of obesity and diabetes, whereas women are more likely to choose white meat and plant-based proteins, potentially reducing these health risks. Additionally, variations in dietary patterns—such as the differences in nutrient intake and health outcomes between vegetarians and traditional meat-eaters—highlight that the relationship between meat consumption and health is dynamic, rather than fixed.

There are recommended limits for meat consumption as part of a healthy diet. The World Cancer Research Fund (WCRF) advises that daily intake of red meat should not exceed 80 g, and processed meat should be minimized. In 2015, the International Agency for Research on Cancer (IARC) of the World Health Organization (WHO) classified processed meat as “carcinogenic to humans” (Group 1) and fresh red meat as “probably carcinogenic to humans” (Group 2 A) (Domingo & Nadal, 2017). Numerous studies have demonstrated that high consumption of red and processed meats is closely linked to the incidence of various diseases. For instance, there is a strong positive correlation between red meat intake or heme iron consumption and the risk of colorectal cancer (Bastide et al., 2011; de Oliveira Otto et al., 2012; Egeberg et al., 2013; Seiwert et al., 2020). Additionally, connections have been established between red meat and other health issues, including heart disease, rheumatoid arthritis, and type 2 diabetes (Diallo et al., 2016; Kontogianni et al., 2008; Schulze et al., 2003; Song et al., 2004; Zhong et al., 2020). Specifically, studies by Bechthold et al. and Sinha et al. have confirmed that the high content of saturated fats, sodium, and preservatives in red meat may contribute to the development of cardiovascular diseases and metabolic disorders (Bechthold et al., 2019; Sinha et al., 2019). Furthermore, analysis of at least six cohorts has shown that the summary results for daily consumption of 100 g of unprocessed red meat range from no statistical significance to a statistically significant increase in risk: an 11 % increase for stroke and breast cancer, a 15 % increase in cardiovascular mortality, a 17 % increase for colorectal cancer, and a 19 % increase for advanced prostate cancer (Wolk, 2017). Moreover, for those consuming 50 g of processed meat daily, the risk of various diseases was statistically significantly elevated: 4 % for total prostate cancer mortality, 8 % for overall cancer mortality, 9 % for breast cancer, 18 % for colorectal cancer, 19 % for pancreatic cancer, 13 % for stroke, 22 % for cardiovascular mortality, and 32 % for diabetes (Wolk, 2017).

In contrast, the association between white meat and these diseases is either weak or negatively correlated (Alegria-Lertxundi et al., 2022; Becerra-Tomás et al., 2016; Damigou et al., 2022; Lupoli et al., 2021; Ramel et al., 2023). Research by Bergeron et al. and Damigou et al. indicates that white meat, particularly poultry, has a more favorable effect on cardiovascular health, potentially due to lower concentrations of saturated fats and the absence of certain harmful additives found in processed red meat (Bergeron et al., 2019; Damigou et al., 2022). Similarly, a study by Alegria et al. found that white meat consumption did not correlate with an increased risk of colorectal cancer, further supporting the idea that it poses fewer health risks than red meat. Table 2 presents a summary of some studies examining the relationship between red and white meat consumption and the risks of diabetes, cardiovascular diseases, and colorectal cancer. As a result, the impact of different types of meat on health has become a major global concern. To scientifically and rationally explain these phenomena, it is essential to conduct in-depth studies on the underlying mechanisms.

**Table 2:** Summary of prospective studies, cohort studies, case-control studies, and meta-analyses of the association between meat (red and white meat) and risk of chronic disease.

Study, country	Subjects(n)	Sex(Age range)	Type of meat studied	Related disease	Findings	Ref.
Cross-sectional study, Iran	482	F (40–60)	Red meat	Metabolic syndrome risk	Increased red meat consumption is cross-sectionally associated with greater risk of metabolic syndrome and inflammation.	(Azadbakht & Esmaillzadeh, 2009)
Case-control study, Greece	848^a^	M&F	Red meat & white meat	Acute coronary syndrome risk	Increased red meat consumption showed a strong positive association with cardiac disease risk, whereas white meat consumption showed less prominent results.	(Kontogianni et al., 2008)
Cohort study, USA	29,017	F	Red meat	CHD mortality	Red meat and dairy intake are associated with coronary heart disease mortality.	(Kelemen et al., 2005)
Case-control study, Iranian	195	NA	Red meat	Stroke risk	Consumption of red meat was associated with greater odds of having stroke in a group of Iranian adults.	(Saneei et al., 2015)
Case-control study,	10,026	NA	Beef, pork or lamb	CRC risk	The association of red meat with CRC risk was weak and significant only for lamb and pork, but not for beef.	(Saliba et al., 2019)
Prospective cohort study, China	510,048	M&F (30–79)	Red meat, poultry and fish	Liver cancer risk	The results showed that red meat and fish consumption were not significantly associated with liver cancer risk; however, poultry meat consumption was negatively associated with liver cancer risk among rural residents	(Wang et al., 2024)
Meta-analysis	NA	NA	White meat	CVD&T2D risk	The results showed no significant effect of white meat consumption on CVD and T2D outcomes	(Ramel et al., 2023)
Prospective study, USA	60,777	F	Seafood and meat	T2D risk	Increased shellfish intake was associated with an elevated risk of T2D, while no significant associations were seen for other seafood, fatty fish, meat and red meat	(Myrmel et al., 2024)
Prospective cohort, French	116,852	F	Processed red meat	Hypertension&diabetes risk	Relative to PRM, fatty fish was associated with a 15 % lower risk of diabetes and hypertension.	(Thao et al., 2023)
Case-control study	105^a^	NA	Red meat	CRC risk	Red meat intake as a promoter of CRC.	(Ghrouz & El Sharif, 2022)
Prospective study, England	71,252	M&F	Red meat, white meat and processed meat	Mortality	Red and processed meat intakes were associated with modest increases in total mortality, cancer mortality, and cardiovascular disease mortality.	(Sinha et al., 2009)
Prospective study	1868	M&F(55–80)	Red meat, white meat and processed meat	Metabolic syndrome	Replacement of Red Meat and Processed Red Meat with White Meat, Fish, Beans, or Eggs Is Associated with a Reduced Risk of Developing MetS.	(Becerra-Tomás et al., 2016)
Cross-sectional study, Brazil	296	M(45.5–55.5)	Red meat and white meat	Metabolic syndrome and insulin resistance	Red but not white meat consumption is associated with metabolic syndrome, insulin resistance and lipid peroxidation.	(Cocate et al., 2015)
Meta-analysis,	NA	NA	White meat	CRC risk	High consumption of total dairy products was associated with a lower CRC risk. However, evidence for fish, white meat, and eggs and the CRC risk were not as strong.	(Alegria-Lertxundi et al., 2022)
Prospective study, USA	29,682	NA	Red meat, white meat and processed meat	CVD risk and all-cause mortality	Processed meat, unprocessed red meat, and poultry intake were significantly associated with new-onset cardiovascular disease (CVD) and all-cause mortality, whereas fish intake was not.	(Zhong et al., 2020)
Prospective study, USA	43,272	M	Red meat	CVD risk	Substituting high quality plant foods for red meat might reduce the risk of CHD.	(Al-Shaar et al., 2020)

Abbreviations: M, male; F, female; CHD, coronary heart disease; CVD, cardiovascular disease; CRC, colorectal cancer; PRM, processed red,meat; MetS, metabolic syndrome; NA, not applicable.

a Number of subjects in case group.

## 3 Heme

### 3.1 Absorption of heme

**Fig. 2:**
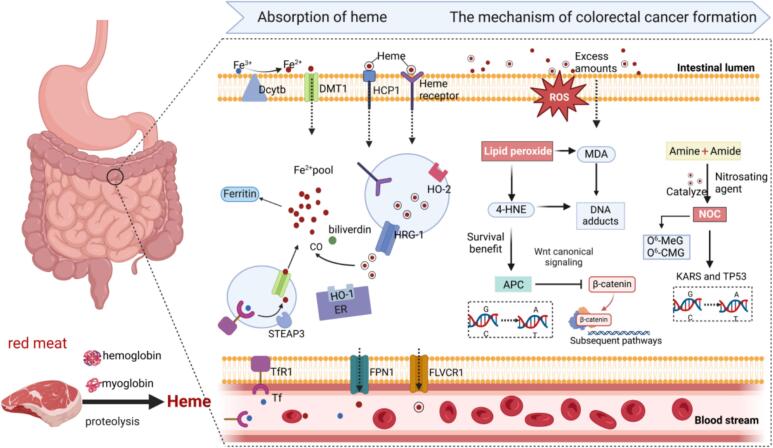
Heme absorption and its mechanism of promoting colorectal carcinogenesis. HCP1, heme carrier protein 1; HO-1, heme oxygenase 1; HRG-1, heme-responsive gene 1; FLVCR1, feline leukemia virus subgroup C receptor 1; Dcytb, cytochrome *b*; DMT1, divalent metal transporter 1; FPN1, ferroportin 1; Tf, transferrin; TfR1, transferrin receptor 1; 4-HNE, 4-hydroxynonenal; MDA, malondialdehyde; NOC, N-nitroso compound; O^6^-CMG, O^6^-carboxymethylguanine; O^6^-MeG, O^6^-methylguanine.

**Fig. 3:**
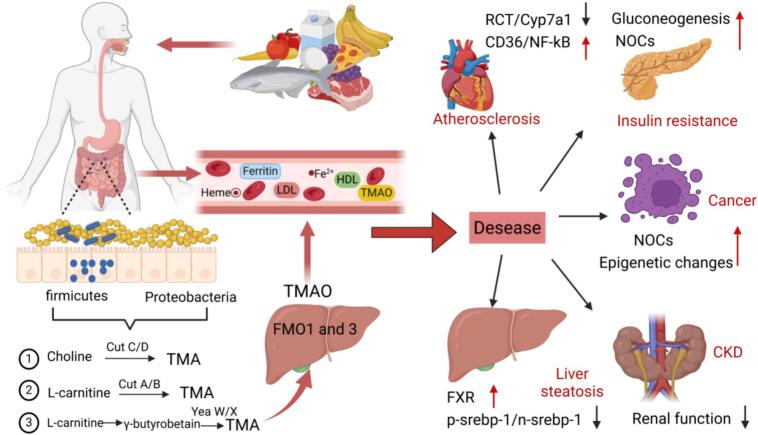
The creation and impact of TMAO. TMA, Trimethylamine; TMAO, trimethylamine N-oxide; FOM1/3, Flavin monooxygease-1/3; RCT, reverse cholesterol transport; Cyp7a1, cholesterol 7-α-hydroxylase; CD36, cluster of differentiation 36; NOC, N-nitroso compound; CKD, chronic kidney disease; FXR, farnesoid X receptor.

Although iron is an essential element for almost all living organisms, excessive iron can be toxic. Al Tappel hypothesized that heme iron in red meat acts as a catalyst for oxidative damage, thereby promoting the development of several chronic diseases (Tappel, 2007). After dietary intake, heme is released from hemoglobin and myoglobin due to the low pH in the stomach and the action of proteolytic enzymes in the stomach and small intestine. The intestinal absorption of heme is thought to occur through two primary mechanisms: receptor-mediated endocytosis and the direct transport of heme by heme carrier protein 1 (HCP1). Once absorbed, heme can be transported from vesicles to the cytoplasm via heme-responsive gene 1 (HRG-1) and subsequently metabolized by heme oxygenase 1 (HO-1) in the endoplasmic reticulum, releasing ferrous ions that contribute to the cytoplasmic iron pool. Some intact heme can be directly released into the bloodstream through the feline leukemia virus subgroup C receptor 1 (FLVCR1), which exports heme into the lumen to protect the cells from heme toxicity when cellular heme levels rise. After ferric iron (Fe^3+^) is reduced to ferrous iron (Fe^2+^) by duodenal cytochrome B (Dcytb), it is transported into the cell by divalent metal transporter 1 (DMT1) and added to the iron pool, where it can either be incorporated into ferritin or transported across the basolateral membrane into the blood via ferroportin 1 (FPN1). In the bloodstream, Fe^3+^ binds to transferrin (Tf) and enters cells with transferrin receptor 1 (TfR1). Inside endosomal vesicles, Fe^3+^ is reduced to Fe^2+^ by the metal reductase STEAP3, and then enters the iron pool via DMT1 (Fig. 2). (See Fig. 3.)

### 3.2 The mechanisms by which heme promotes chronic diseases

Dietary heme is poorly absorbed in the small intestine, with approximately 90 % of dietary heme entering the colon (Young et al., 1989). Therefore, individuals who consume red meat over the long term have their colonic mucosa continually exposed to high concentrations of heme. Compared to other parts of the gastrointestinal tract, the carcinogenic effects of heme are particularly pronounced in the colon (Sesink et al., 2000). Heme iron can generate various DNA-damaging factors, including reactive oxygen species (ROS), lipid peroxidation end-products, and NOCs, all of which contribute to DNA damage and gene mutations, key factors in the development of cancer. Heme iron promotes the production of ROS through the Fenton reaction, and ROS can directly oxidize the bases in DNA, leading to oxidative damage (Stohs & Bagchi, 1995). ROS can also induce DNA double-strand or single-strand breaks, further destabilizing the genome. Additionally, ROS promote lipid peroxidation of unsaturated fatty acids, with major lipid peroxidation products being reactive aldehydes, such as malondialdehyde (MDA) and 4-hydroxynonenal (4-HNE). MDA and 4-HNE can form mutagenic DNA adducts, further inducing gene mutations in the intestinal epithelium (Guéraud et al., 2010). NOCs are formed under the influence of nitrosating agents, which nitrosate amines and amides produced by bacterial decarboxylation of amino acids. NOCs are alkylating agents, and studies have shown that red meat (rather than white meat) intake dose-dependently promotes the endogenous formation of NOCs, as evidenced by increased apparent total NOC (ATNC) concentrations in feces (Hughes et al., 2001). NOC compounds have the ability to interact with the DNA of colon cells, leading to the formation of mutagenic substances like O^6^-methylguanine (O^6^-MeG) and O^6^-carboxymethylguanine (O^6^-CMG). These unrepaired O^6^-MeG and O^6^-CMG undergo mismatches, leading to (G → A) mutations. Furthermore, the combination of NOCs and reactive oxygen species can also promote (G → T) mutations in genes (Shibutani et al., 1991). APC, KRAS, and TP53 are key tumor suppressor genes and oncogenes, with mutations in these genes frequently serving as early indicators of cancer onset. TP53 gene mutations lead to cell cycle dysregulation and inhibition of apoptosis, while KRAS mutations promote abnormal cell proliferation. APC is a key effector molecule that controls β-catenin degradation, and loss of its activity results in uncontrolled cell proliferation in the colonic epithelium (Markowitz & Bertagnolli, 2009; Schneikert & Behrens, 2007). Compared to normal colonic cells, the 4-HNE produced in the intestine can provide a survival advantage to APC-mutated colonic cells, “selecting” precancerous cells and increasing the mutation frequency in normal cells. Therefore, it can be hypothesized that heme and its metabolic byproducts promote colorectal cancer by inducing mutations in these genes (Fig. 2).

Additionally, accumulating evidence indicates that heme-induced hyperproliferation of colonic epithelial cells correlates with heme-dependent gut microbiota alterations. Dietary heme increases Akkermansia abundance and enhances sulfate-reducing bacteria (SRB) activity, promoting intestinal mucus layer degradation. This erosion directly exposes colonic epithelium to luminal cytotoxic compounds and enteric pathogens—including bacterial lipopolysaccharides (LPS) (Ijssennagger et al., 2015). Pathogens reaching the epithelium trigger immune responses that exacerbate intestinal inflammation and promote colorectal carcinogenesis. Mechanistically, heme iron activates Toll-like receptor 4 (TLR4), driving nuclear factor κB (NF-κB) translocation and upregulating pro-inflammatory cytokines (TNF-α, IL-6). In animal studies, high-dose heme iron (100 mg/kg) elevated colonic TNF-α concentrations 2.8-fold and significantly increased expression of macrophage infiltration marker F4/80 (Wei et al., 2024). Heme iron polarizes macrophages toward the M1 phenotype, inducing reactive nitrogen species (RNS) release that causes DNA damage. Concurrently, it activates the NLRP3 inflammasome, facilitating caspase-1-mediated IL-1β maturation and accelerating chronic inflammation-to-carcinoma transition. Collectively, heme-driven hyperproliferation links to dysbiosis-impaired mucosal barrier function, increasing epithelial exposure to cytotoxic compounds.

In addition to colorectal cancer, heme intake is also closely associated with type 2 diabetes and its major complication, cardiovascular disease (CVD) (Jin et al., 2024). Human iron homeostasis is maintained through the regulation of iron absorption, intracellular utilization, and storage, ensuring that transferrin saturation remains at physiological levels. Less than 10 % of the body's iron requirements are met through intestinal absorption, with excess iron being excreted via FPN1 (Fig. 2) (Soe-Lin et al., 2009). Hepcidin is a peptide hormone secreted by liver cells that regulates systemic iron flow by mediating the internalization and lysosomal degradation of FPN1 (Nemeth et al., 2004). Iron may play a role in the development of diabetes and cardiovascular disease through several mechanisms. On one hand, the expression of antioxidant enzymes in the pancreas is low, and since iron is a potent pro-oxidant and catalyst, it may increase oxidative stress, leading to damage of pancreatic β-cells and subsequently interfering with insulin synthesis and secretion (Reddy & Clark, 2004). On the other hand, reactive oxygen species can oxidize low-density lipoprotein, promoting cell necrosis and apoptosis, thereby exacerbating atherosclerosis and endothelial damage (Li & Zhang, 2021). Additionally, iron deposition can impair the liver, adipose, and muscle tissues' response to insulin, inducing insulin resistance. Excessive iron accumulation in the liver disrupts glucose metabolism, resulting in hyperinsulinemia, primarily due to reduced insulin extraction and impaired insulin signaling (Niederau et al., 1984). In atherosclerotic lesions, monocytes, macrophages, endothelial cells, vascular smooth muscle cells, and platelets all show iron overload, and their involvement promotes the progression of atherosclerosis (Vinchi et al., 2014). In in vitro models, incubating rat adipocytes with excess iron results in reduced insulin-stimulated glucose transport and increased lipolysis (Rumberger et al., 2004). Iron homeostasis disruption also leads to changes in various adipokine concentrations, such as leptin and adiponectin, ultimately causing lipid metabolism disorders and insulin resistance. Some evidence suggests that iron overload may also affect skeletal muscle metabolism, and reduced heme synthesis, along with dysregulated iron uptake or output, may contribute to abnormal glucose metabolism in aging muscle (Milford et al., 2019).

## 4 Pathogenesis of saturated fatty acids

Excessive intake of saturated fatty acids (SFAs) is a major factor in the development of cardiovascular disease and insulin resistance (Boden, 2003; Boden & Salehi, 2013). Studies have found that SFAs, particularly palmitic acid (C16:0), can activate key negative regulators in the insulin signaling pathway, inhibiting the phosphorylation of IRS-1, thereby lessing the effects of insulin (Hotamisligil, 1999). The accumulation of SFAs also inhibits mitochondrial β-oxidation, leading to elevated concentrations of free fatty acids (FFAs), which in turn trigger insulin resistance (Kelley et al., 1999). Excessive SFA intake also increases liver fat deposition, contributing to non-alcoholic fatty liver disease (NAFLD), which exacerbates the pathological progression of type 2 diabetes mellitus (T2DM).

Cardiovascular disease (CVD) is a leading cause of death in developed countries, encompassing conditions such as coronary heart disease (CHD), stroke, and myocardial infarction (MI). In most cases, the underlying cause of CVD is atherosclerosis, which involves the gradual thickening of the arterial walls and loss of elasticity due to the accumulation of lipids (mainly cholesterol esters), connective tissue, and later, calcium (Schaefer & Brousseau, 1998). Ancel Keys proposed that a high dietary fat intake, particularly saturated fatty acids (SFAs), is closely associated with elevated concentrations of total cholesterol (TC) and low-density lipoprotein cholesterol (LDL-C) in the serum, thus significantly increasing the risk of cardiovascular diseases (Keys, 1953; Willett, 2012). However, natural low-density lipoprotein (LDL) does not directly initiate the mechanisms of atherosclerosis; it must undergo oxidative modification to become pathogenic (Quinn et al., 1985). When LDL particles penetrate the damaged endothelial layer and enter the intima of the vessel wall, they are oxidized by reactive oxygen species (ROS) and transformed into oxidized LDL (Ox-LDL) (Carnevale et al., 2014). This process causes Ox-LDL to accumulate in the subendothelial space, activating monocytes to migrate into the vessel wall, where they differentiate into macrophages. After macrophages engulf Ox-LDL, they gradually accumulate and transform into foam cells, which exhibit the characteristic “soap bubble” morphology, ultimately leading to the formation of atherosclerotic plaques. These plaques restrict blood flow to the heart, thereby increasing the risk of cardiovascular diseases (Haberland et al., 2001; Palinski et al., 1989). On the other hand, diets rich in monounsaturated fatty acids and ω-3 polyunsaturated fatty acids have shown potential to effectively increase high-density lipoprotein (HDL) concentrations. HDL plays a crucial role in delaying the oxidation of LDL and promoting cholesterol efflux, preventing its transformation into foam cells (Miller et al., 1998; Schaefer & Brousseau, 1998). This cholesterol efflux process actually serves as a primary defense against the transformation of macrophages into foam cells. Additionally, HDL helps prevent smooth muscle cell (SMC) migration and proliferation induced by cytokines and growth factors, effectively inhibiting platelet migration and aggregation in the plaque area, thereby providing additional protection for cardiovascular health.

## 5 Pathogenesis of trimethylamine N-oxide

Red meat is rich in carnitine, whereas white meat contains very little of such compounds. As a significant source of trimethylamine-N-oxide (TMAO), red meat negatively impacts both the gut microbiota and host health (Wang et al., 2019). Substances such as choline, l-carnitine, and phosphatidylcholine are converted into trimethylamine (TMA) by the gut microbiota, which is then released into the bloodstream. TMA is subsequently further oxidized in the liver by flavin-containing monooxygenase (FMO) enzymes to form TMAO (Cashman et al., 2001). TMAO has been linked to a variety of diseases, including diabetes, atherosclerosis, hypertension, chronic kidney disease, gastrointestinal cancer, hepatic steatosis, and Alzheimer's disease (Oellgaard et al., 2017; Tan et al., 2019). A cross-sectional study showed that plasma TMAO concentrations were significantly higher in patients with coronary heart disease compared to healthy subjects (Dong et al., 2018). TMAO disrupts liver bile acid synthesis by inhibiting cholesterol 7-α-hydroxylase (CYP7A1), as bile acids regulate lipid metabolism via activation of the farnesoid X receptor. Therefore, inhibiting CYP7A1 affects bile acid's ability to eliminate cholesterol and other lipids, leading to the accumulation of total cholesterol, low-density lipoprotein, and triglycerides, which promotes hepatic fat accumulation and the formation of atherosclerotic plaques (Tang et al., 2013). In cellular studies, TMAO has been shown to enhance macrophage uptake of cholesterol by increasing the surface expression of macrophage scavenger receptors (CD36), thereby stimulating macrophage oxidation and promoting the progression of atherosclerotic plaques (Wang et al., 2011). TMAO also increases platelet activity via Ca^2+^ stimulation and contributes to atherosclerosis, further promoting the development of cardiovascular disease (CVD) (Zhu et al., 2016).

Moreover, TMAO contributes to the progression of diabetes and insulin resistance through stimulation of gluconeogenesis. Since TMA is a precursor for NOC formation, high TMAO concentrations correlate with elevated NOC concentrations, which are involved in DNA damage, lipid peroxidation, oxidative stress, and the activation of inflammatory pathways leading to insulin resistance (Oellgaard et al., 2017). Animal studies have shown that high-fat diets or supplementation with choline or TMAO induce renal tubular interstitial fibrosis and promote the expression of kidney injury biomarkers and pro-fibrotic genes, resulting in structural kidney damage (Tang et al., 2015). Overall, TMAO negatively impacts health through multiple pathways, particularly in cardiovascular diseases and metabolic disorders. Although the specific mechanisms are still being investigated, understanding these mechanisms is crucial for developing more effective prevention and treatment strategies.

## 6 Others

In addition to the substances mentioned earlier, meat contains various bioactive compounds, which are typically present in higher amounts in red meat than in white meat and are closely associated with the onset of several chronic diseases (Li et al., 2018). For example, red meat is rich in purines, which are metabolized in the body to form uric acid. Excessive accumulation of uric acid can lead to gout. Furthermore, elevated uric acid concentrations place additional stress on the kidneys, and prolonged hyperuricemia not only increases the risk of kidney disease but can also lead to kidney stones or even kidney failure (Fathallah-Shaykh & Cramer, 2014). Some studies suggest that a high purine diet is significantly associated with an increased risk of cardiovascular diseases and the onset of metabolic syndrome, potentially by raising uric acid concentrations and triggering systemic inflammation, thereby affecting heart health and vascular function (Yanai et al., 2021). Neu5Gc, a sialic acid derivative, can promote tumor growth and spread by binding to receptors on tumor cells, thus driving tumor development and metastasis. After consuming Neu5Gc, the body's immune system recognizes it as a foreign substance, triggering an immune response. This response may result in chronic inflammation, further promoting the onset of cardiovascular diseases, diabetes, and other chronic conditions. Particularly when Neu5Gc accumulates in the vascular endothelium, kidneys, or other organs, it can exacerbate tissue damage, leading to long-term chronic health issues that adversely affect overall health (Liang et al., 2024).

## 7 Summary and dietary recommendations

Dietary meat has long been an essential component of the human diet, providing a rich source of protein, energy, minerals, and vitamins that are crucial for human health. Due to differences in composition, red meat and white meat have distinct effects on human health. Red meat generally contains higher amounts of saturated fat and heme iron, and excessive long-term consumption has been closely linked to the development of chronic diseases such as cardiovascular disease, diabetes, kidney disease, and certain types of cancer. In contrast, white meat, such as fish and poultry, typically has lower fat content and is rich in unsaturated fatty acids, vitamins, and minerals, often making it a healthier choice. In Europe, where red meat consumption is relatively high, there is a significant association between red meat intake and the prevalence of related diseases. Therefore, dietary recommendations regarding meat consumption from several European countries are summarized in Table 3 (Cocking et al., 2020). Although dietary guidelines differ among countries, there is a general consensus to reduce the intake of red meat and processed meat, especially since excessive consumption of processed meat has been linked to multiple health issues. In contrast, it is recommended to replace red meat with alternatives like fish and poultry, which not only provide high-quality protein but also help reduce the risk of chronic diseases. Additionally, the Food and Agriculture Organization (FAO) of the United Nations has developed a global Food-Based Dietary Guidelines (FBDG) database, providing scientific support for national dietary policies and recommendations (Food and Agriculture Organization (2024).

**Table 3:** Meat consumption guidelines in Europe.

Country/Organization	Guidance on meat consumption	Additional comments	Websites	Reference
Albania	Adults should consume 1 portion of meat, fish, egg or cheese alternatively every day (1 portion of meat =100–120 g).	Guidance also available for children and adolescents	https://www.fao.org/nutrition/education/food-dietary-guidelines/regions/countries/albania/en/	(Albanian Department Of Public Health, 2008)
Austria	Eat up to 3 servings of lean meat or low-fat sausages per week(300–450 g).	Eat red meat and sausages in moderation	https://www.fao.org/nutrition/education/food-dietary-guidelines/regions/countries/austria/en/	(Austrian Ministry Of Health And The National Nutrition Commission, 2010)
Belgium	Do not eat more than 75–100 g/d of meat, fish, eggs or products made with these foods.	Not applicable	https://www.fao.org/nutrition/education/food-dietary-guidelines/regions/countries/belgium/en/	(Living, Flemish Institute, and Healthy, 2005)
Bulgaria	Consume poultry meat without the skin and lean red meats up to 3 times per week (100 g/serving).	Replace meat and meat products often with fish, poultry or pulses	https://www.fao.org/nutrition/education/food-dietary-guidelines/regions/countries/bulgaria/en/	(Bulgarian National Center ofPublic Health Protection (NCPHP), 2006)
Croatia	Choose lean meats (for example, poultry, rabbit) and fish over red meat.	Not applicable	https://www.fao.org/nutrition/education/food-dietary-guidelines/regions/countries/croatia/en/	(Ministry of Health (Republic of Croatia), 2002)
Denmark	Choose lean meats and cold meats.	Not applicable	https://www.fao.org/nutrition/education/food-dietary-guidelines/regions/countries/denmark/en/	(Ministry of Food, Agriculture and Fisheries (Denmark), 2013)
Estonia	Eat less red meat, prefer fish and poultry.	Not applicable	https://www.fao.org/nutrition/education/food-dietary-guidelines/regions/countries/estonia/en/	(Estonian National Institute for Health Development, 2017)
France	Eat meat, fish, other seafood and eggs alternating 1 or 2 times per d.	Limit red meat to <500 g/week and processed meat to<25 g/d.	https://www.fao.org/nutrition/education/food-dietary-guidelines/regions/countries/france/en/	(French Agency for Food Environmental and Occupational Health and Safety (ANSES), 2016)
Georgia	Eat 1–3 portions per d of cooked lean meat, poultry, fish, eggs or legumes (1 portion of meat = 80 g).	Replace fatty meat and meat products by legumes, fish and chicken and low-fat meat	https://www.fao.org/nutrition/education/food-dietary-guidelines/regions/countries/georgia/en/	(National Centre for Disease Control and Public Health (Georgia), 2005)
Germany	Eat meat, sausages and eggs in moderation.	Not applicable	https://www.fao.org/nutrition/education/food-dietary-guidelines/regions/countries/germany/en/	(German Nutrition Society, 2013)
Greece	Consumption of poultry, eggs and red meat should not exceed on the average 1 serving per d (1 serving of cooked lean meat = 60 g).	Poultry is much preferred over red meat	https://www.fao.org/nutrition/education/food-dietary-guidelines/regions/countries/greece/en/	(Medicine, Greek Institute For Preventive, 2014)
Ireland	Consume two servings of lean meat, poultry, fish, eggs, beans or nuts per d.	Limit the consumption of processed meat	https://www.fao.org/nutrition/education/food-dietary-guidelines/regions/countries/ireland/en/	(Food Safety Authority of Ireland (FSAI), 2011)
Latvia	Eat legumes, fish or lean meat. The recommended weekly amount of lean meat is 300–600 g.	Guidance also available for children, adolescents and the elderly	https://www.fao.org/nutrition/education/food-dietary-guidelines/regions/countries/latvia/en/	(Latvia, 2008)
Malta	Eat 2 servings per d of lean white meat (1 serving = 100 g). Limit consumption of red meat to <2 servings twice weekly(1 serving = 90 g).	If desired, occasionally consume small quantities of processed meat	https://www.fao.org/nutrition/education/food-dietary-guidelines/regions/countries/malta/en/	(Directorate, 2016)
UK	If you eat more than 90 g of red or processed meat per d, try to cut down to no more than 70 g per d.	Choose lean cuts of meat and grill meat instead of frying	https://www.fao.org/nutrition/education/food-dietary-guidelines/regions/countries/united-kingdom/en/	(Public Health England, 2016)
World Cancer Research Fund	Limit red meat consumption to <500 g/week.	Eat very little, if any, processed meat	https://pubmed.ncbi.nlm.nih.gov/18452640/	(Wiseman, 2008)

Based on our analysis, we propose the following specific recommendations: red meat consumption should be limited to less than 350 g per week, and processed meat ought to be avoided. It is also advisable to prioritize white meat—such as poultry and fish—and incorporate more plant-based protein sources into one's diet. Healthier cooking methods, including steaming and stewing, are encouraged over high-temperature grilling or frying to reduce the formation of harmful compounds. Moreover, dietary diversity should be emphasized through increased consumption of fruits, vegetables, and whole grains, as these foods are rich in fiber, antioxidants, and micronutrients that help mitigate oxidative stress and lower the risk of chronic diseases.

In conclusion, our findings underscore the necessity of moderating red meat consumption and replacing it with healthier alternatives to lower the burden of chronic diseases. Public health strategies should promote dietary patterns that are not only balanced and diverse but also practical and culturally adaptable. These evidence-based adjustments align with global sustainability and health objectives, supporting both individual well-being and broader nutritional guidelines.

## CRediT authorship contribution statement

**Huiling Zhang:** Writing – original draft. **Mailin Gan:** Writing – review & editing. **Qihang Wu:** Writing – review & editing. **Haifeng Dan:** Writing – review & editing. **Lei Chen:** Funding acquisition. **Lili Niu:** Writing – review & editing. **Ye Zhao:** Writing – review & editing. **Yan Wang:** Resources. **Li Zhu:** Funding acquisition. **Linyuan Shen:** Writing – review & editing.

## Declaration of competing interest

The authors declare that the research was conducted in the absence of any commercial or financial relationships that could be construed as a potential conflict of interest.

## Data Availability

Data will be made available on request.
